# Leveraging brain cortex-derived molecular data to elucidate epigenetic and transcriptomic drivers of complex traits and disease

**DOI:** 10.1038/s41398-019-0437-2

**Published:** 2019-02-28

**Authors:** Charlie Hatcher, Caroline L. Relton, Tom R. Gaunt, Tom G. Richardson

**Affiliations:** MRC Integrative Epidemiology Unit, Population Health Sciences, Bristol Medical School, University of Bristol, Bristol, UK

## Abstract

Integrative approaches that harness large-scale molecular datasets can help develop mechanistic insight into findings from genome-wide association studies (GWAS). We have performed extensive analyses to uncover transcriptional and epigenetic processes which may play a role in complex trait variation. This was undertaken by applying Bayesian multiple-trait colocalization systematically across the genome to identify genetic variants responsible for influencing intermediate molecular phenotypes as well as complex traits. In this analysis, we leveraged high-dimensional quantitative trait loci data derived from the prefrontal cortex tissue (concerning gene expression, DNA methylation and histone acetylation) and GWAS findings for five complex traits (Neuroticism, Schizophrenia, Educational Attainment, Insomnia and Alzheimer’s disease). There was evidence of colocalization for 118 associations, suggesting that the same underlying genetic variant influenced both nearby gene expression as well as complex trait variation. Of these, 73 associations provided evidence that the genetic variant also influenced proximal DNA methylation and/or histone acetylation. These findings support previous evidence at loci where epigenetic mechanisms may putatively mediate effects of genetic variants on traits, such as *KLC1* and schizophrenia. We also uncovered evidence implicating novel loci in disease susceptibility, including genes expressed predominantly in the brain tissue, such as *MDGA1*, *KIRREL3* and *SLC12A5*. An inverse relationship between DNA methylation and gene expression was observed more than can be accounted for by chance, supporting previous findings implicating DNA methylation as a transcriptional repressor. Our study should prove valuable in helping future studies prioritize candidate genes and epigenetic mechanisms for in-depth functional follow-up analyses.

## Background

Genome-wide association studies (GWAS) have been integral in identifying thousands of genetic variants associated with complex traits and disease. The vast majority of genetic variants identified in these studies reside in intergenic or intronic regions of the genome and are therefore predicted to exert their effects on complex traits via changes in gene regulation^1^. Furthermore, there is evidence which suggests that GWAS hits are often located within regions of open chromatin and enhancers^2^. Typically, genetic variants associated with molecular phenotypes are enriched amongst single-nucleotide polymorphisms (SNPs) that are linked to traits and diseases^3^. Such variants are known as quantitative trait loci (QTL) and can affect molecular phenotypes such as: gene expression (eQTL), and epigenetic mechanisms including DNA methylation (mQTL) and histone acetylation (haQTL). DNA methylation and histone acetylation are alterations that affect gene expression without altering the DNA sequence. Several genetic variants have been identified that occur in the same genomic region and influence both gene expression and DNA methylation. In these cases, it is possible that the eQTL and mQTL share a common causal variant (CCV)^4^.

Several post-GWAS approaches exist to help functionally characterize non-coding variants^5–7^. In particular, there has been an emphasis on integrating eQTL and GWAS data together, which can be valuable in terms of identifying the underlying genes responsible for associations detected by GWAS. Recently, similar endeavours have extended the scope of their analysis to also evaluate additional molecular phenotypes (e.g. mQTL and haQTL) as well as gene expression^8–11^. A novel method in this paradigm involves calculating approximate Bayes factors^12^ to assess the likelihood that the genetic variant responsible for an association with a complex trait is also responsible for influencing intermediate molecular phenotypes (i.e. the likelihood they share a CCV). This multiple-trait colocalization (moloc) method has been shown to help characterize GWAS loci and develop mechanistic insight into the causal pathway from genetic variant to complex trait^13^. Furthermore, inclusion of an additional molecular trait into the analysis (e.g. complex trait, gene expression and DNA methylation vs. complex trait and gene expression alone) has been shown to increase power and assist in identifying novel disease susceptibility loci^13^.

The recent large influx of tissue-specific molecular data provides an unprecedented opportunity to assess the functional relevance of GWAS hits. Recently, a resource has become available that comprises QTL data derived from the dorsolateral prefrontal cortex in up to 494 subjects^14^. Brain xQTL Serve provides a list of SNPs associated with gene expression, DNA methylation and/or histone modifications specific to the same brain region^14^. Whilst progress has been made in terms of identifying genetic variants influencing neurological phenotypes and diseases, not enough is known about the biological effects of genetic risk factors. In this study, we have jointly analysed genetic variants identified across GWAS of five complex traits and diseases alongside the variants listed in the Brain xQTL Serve resource. In doing so, we aim to identify CCVs for complex traits and gene expression, and where possible, DNA methylation and histone acetylation. We selected a range of neurological, psychiatric, personality and behavioural traits (Neuroticism, Schizophrenia, Educational Attainment, Insomnia and Alzheimer’s disease) with publicly available large GWAS summary statistics, where we believed QTL data specific to the brain may be particularly biologically relevant. Uncovering evidence that epigenetic factors reside on the causal pathway along with gene expression can be extremely valuable for disease prevention due to early diagnosis. Additionally, the use of genetic evidence can be beneficial for identifying and selecting drug targets^15^.

## Methods

### Genome-wide association studies

We obtained summary statistics from five independent GWAS for the following complex traits: Neuroticism (*n* = 274,108)^16^, Schizophrenia (cases = 35,467, controls = 46,839)^17^, Educational Attainment (*n* = 293,723)^18^, Alzheimer’s disease (cases = 17,008, controls = 37,154)^19^ and Insomnia (*n* = 336,965)^16^. Information on all GWAS datasets can be found in Supplementary Table 1. Linkage disequilibrium (LD) clumping was undertaken using PLINK v1.9^20^ with a reference panel consisting of European (CEU) individuals from phase 3 (version 5) of the 1000 genomes project^21^. This allowed us to identify the top independent loci for each set of results based on the conventional GWAS threshold (*P* < 5.0 × 10^−08^).

### Brain tissue-derived QTL for three molecular phenotypes

All QTL data used in this study were obtained from the Brain xQTL Serve resource^14^. Genotype data in this resource was generated from 2093 individuals of European descent from the Religious Orders Study (ROS and Memory and Aging Project study cohorts (http://www.radc.rush.edu/). Gene expression (RNA-sequencing (RNA-seq); *n* = 494), DNA methylation (450 K Illumina array; *n* = 468) and histone modification (H3K9Ac ChIP-seq; *n* = 433) data were derived from the dorsolateral prefrontal cortex of post-mortem samples. eQTL were based on 13,484 expressed genes, mQTL on 420,103 methylation sites and haQTL on 26,384 acetylation domains. eQTL and haQTL results were available for variants within 1 Mb of their corresponding probes, whereas mQTL results were restricted to a 5 kb window^14^.

### Gene-centric multiple-trait colocalization

We extracted effect estimates for all variants within 1 Mb of the lead SNP (i.e. *P* < 5.0 × 10^–08^) for each clumped region using results from each of the five GWAS. *P* values for molecular QTL were then extracted for the same set of SNPs using the Brain xQTL resource. Loci residing within the major histocompatibility complex region (chr6: 25−35 Mb) were removed due to extensive LD within this region, which may result in false-positive findings. The moloc method was then used to assess the likelihood that the variant at each region responsible for variation in complex traits was also responsible for influencing the expression of a nearby gene (i.e. within a 1 Mb distance of the lead GWAS SNP). As demonstrated previously^13^, we simultaneously investigated whether variants responsible for both gene expression and complex trait variation may also influence proximal epigenetic traits in a gene-centric manner. However, unlike previous work, which evaluated three traits at a time, we have investigated up to four traits in each analysis (i.e. complex trait, gene expression, DNA methylation and histone acetylation).

To achieve this, we used coordinates from Ensembl^22^ to map CpG sites and histone peaks to genes using a 50-kb window upstream and downstream of each gene. We then ran moloc to assess all gene–CpG–histone combinations within each region of interest. Summed posterior probabilities were computed for all scenarios where GWAS trait and gene expression colocalized. The reason for this is because if epigenetic mechanisms are responsible for mediating the effect of genetic variants on complex traits, then we would expect gene expression to also reside on this causal pathway. Therefore, 10 scenarios were considered of interest: GE, GE,M, GE,H, GE,M,H, GEM, GEM,H, GEH, GEH,M, GEMH, where evidence of a shared causal variants for GWAS complex traits is defined as ‘G', gene expression as ‘E’, DNA methylation as ‘M’ and histone acetylation as ‘H’. The ‘,’ denotes a scenario where there is a different causal variant for each molecular phenotype. For example, GE,M would represent a situation where the same causal variant is shared between the GWAS trait and gene expression, but a different causal variant for DNA methylation.

As recommended by the authors of moloc^13^, a summed posterior probability of association (PPA) ≥80% for these 10 scenarios was considered strong evidence that a genetic variant was responsible for changes in both molecular phenotype(s) and complex trait variation. Therefore, a GEMH scenario with a posterior probability ≥80% would represent a case where there is evidence that GWAS trait, gene expression, DNA methylation and histone acetylation colocalize and share a causal variant. When a gene–trait combination provided evidence of colocalization with multiple CpG sites or histone peaks, we only reported the association for the combination with the highest PPA. This was to reduce the number of findings detected due to co-methylation/probes within the same histone peak that were measuring the same epigenetic signatures.

Regions with fewer than 50 common SNPs (minor allele frequency ≤5%) were not considered in the moloc analysis in order to reduce the number of spurious findings. Prior probabilities of 1 × 10^−04^, 1 × 10^−06^, 1 × 10^−07^ and 1 × 10^−08^ were used in all analyses, which was also recommended by the authors of moloc. Furthermore, we used the option to adjust Bayes factors for overlapping samples as this was the case for the xQTL datasets. Manhattan plots to illustrate findings were subsequently generated using code adapted from the ‘qqman’ package^23^.

### Identifying potentially novel loci in disease susceptibility

We also applied our analytical pipeline as described above to independent GWAS loci with *P* values between the conventional threshold (*P* < 5.0 × 10^−08^) and *P* ≤ 1.0 × 10^−06^. All parameters were the same as in the previous analysis. We hypothesized that incorporating additional evidence on molecular phenotypes could help to elucidate potentially novel loci, which are likely to be identified as sample sizes of future GWAS increase. Although the observed effects of these loci on traits alone do not meet the conventional GWAS threshold, we took evidence of colocalization (again defined as a combined PPA ≥80%) at these loci as novel evidence implicating them in disease, which can be used to prioritize them for future evaluation.

### Functional informatics

#### Pathway analysis

For all scenarios where GWAS trait and gene expression colocalize based on a combined PPA of ≥80%, we compiled a list of associated genes for each trait. Where multiple genes at a region provided a PPA ≥80% for the same GWAS SNP, we took forward the gene with the highest PPA. Pathway analysis was then undertaken with a gene list for each complex trait using ConsensusPathDB^24^. This was to investigate whether multiple associated genes in our analysis reside along established biological pathways more than we would expect by chance.

#### Tissue-specific analysis

We also investigated whether any genes detected in our analysis were predominantly expressed in the brain tissue using three RNA-seq datasets: the Human Protein Atlas (HPA)^25^, the Genotype-Tissue Expression project (GTEx)^26^ and the Mouse ENCODE project^27^. We used the ‘TissueEnrich’ R Package to identify evidence of enrichment based on three definitions:^25^

- *Tissue enriched***:** Genes with an expression level ≥1 TPM (transcripts per million) or FPKM (fragments per kilobase of exon model per million reads mapped) plus at least 5-fold higher expression levels in a particular tissue when compared to all other tissues.
- *Group enriched***:** Genes with an expression level ≥1 TPM or FPKM plus at least 5-fold higher expression levels in a group of 2–7 tissues when compared to other tissues not considered to be ‘Tissue enriched’.
- *Tissue enhanced***:** Genes with an expression level ≥1 TPM or FPKM plus at least 5-fold higher expression levels in a particular tissue compared to the average levels in all other tissues, and not considered to be either ‘Tissue enriched’ or ‘Group enriched’.

Evidence of enrichment under the ‘Tissue enriched’ definition therefore suggests that a gene is specifically expressed in a single tissue type. This suggests that associations between this gene and a complex trait may be tissue specific (i.e. they would only be detected using data derived from one specific tissue). Enrichment based on the ‘Group enriched’ definition suggests that associations may be detected in a set of tissue types (i.e. 2–7 different types), which are expressed at least 5-fold higher than all other tissues considered. Evidence detected using the ‘Tissue enhanced’ definition suggests that this gene is more ubiquitously expressed (in more than seven different tissues), although expression in a specific tissue type is considerably higher than the others (5-fold higher compared to the average of all others). As such, genes detected using this definition may be strongly dependent on using data derived from one specific tissue, although associations may still be detected in other tissue types (i.e. they are not ‘tissue specific’).

For each dataset, we were only interested in genes predominantly expressed in the brain tissue, that is, ‘Cerebral Cortex’ in HPA, ‘Brain’ in GTEx and ‘Cerebellum’, ‘Cortex’ or ‘E14.5 Brain’ in the Mouse ENCODE project. Heatmaps to illustrate enrichment across all possible tissues from these datasets were generated using the ‘ggplot’ R package’^28^.

#### Orienting directions of effect between molecular traits and regulatory region annotation

We oriented the direction of effect between transcriptional and epigenetic traits for detected associations: firstly, between gene expression and DNA methylation and then between gene expression and histone acetylation. For associations with evidence of colocalization between the two traits being assessed, we evaluated whether the lead SNP was correlated with molecular traits in the same direction using coefficients from the xQTL resource. We applied the hypergeometric test to investigate whether there was an enrichment of a particular direction of effect between molecular traits more than we would expect by chance. Background expectations were calibrated using randomly selected lead SNPs across the genome that were associated with both proximal gene expression and DNA methylation (*P* < 1.0 × 10^−04^). Permutation testing was applied for 10,000 iterations by sampling the same number of SNPs being evaluated.

Lastly, we obtained regulatory data from the Roadmap Epigenetics Project^29^ from 10 different types of the brain tissue. We used BEDtools^30^ to evaluate whether lead SNPs, CpG sites and histone peaks with evidence of colocalization from our study reside within promoters, enhancers and histone marks using these datasets. All analyses in this study were undertaken using R (version 3.3.1).

## Results

### Colocalization between gene expression, DNA methylation and histone acetylation at risk loci for five complex traits

**Fig. 1:**
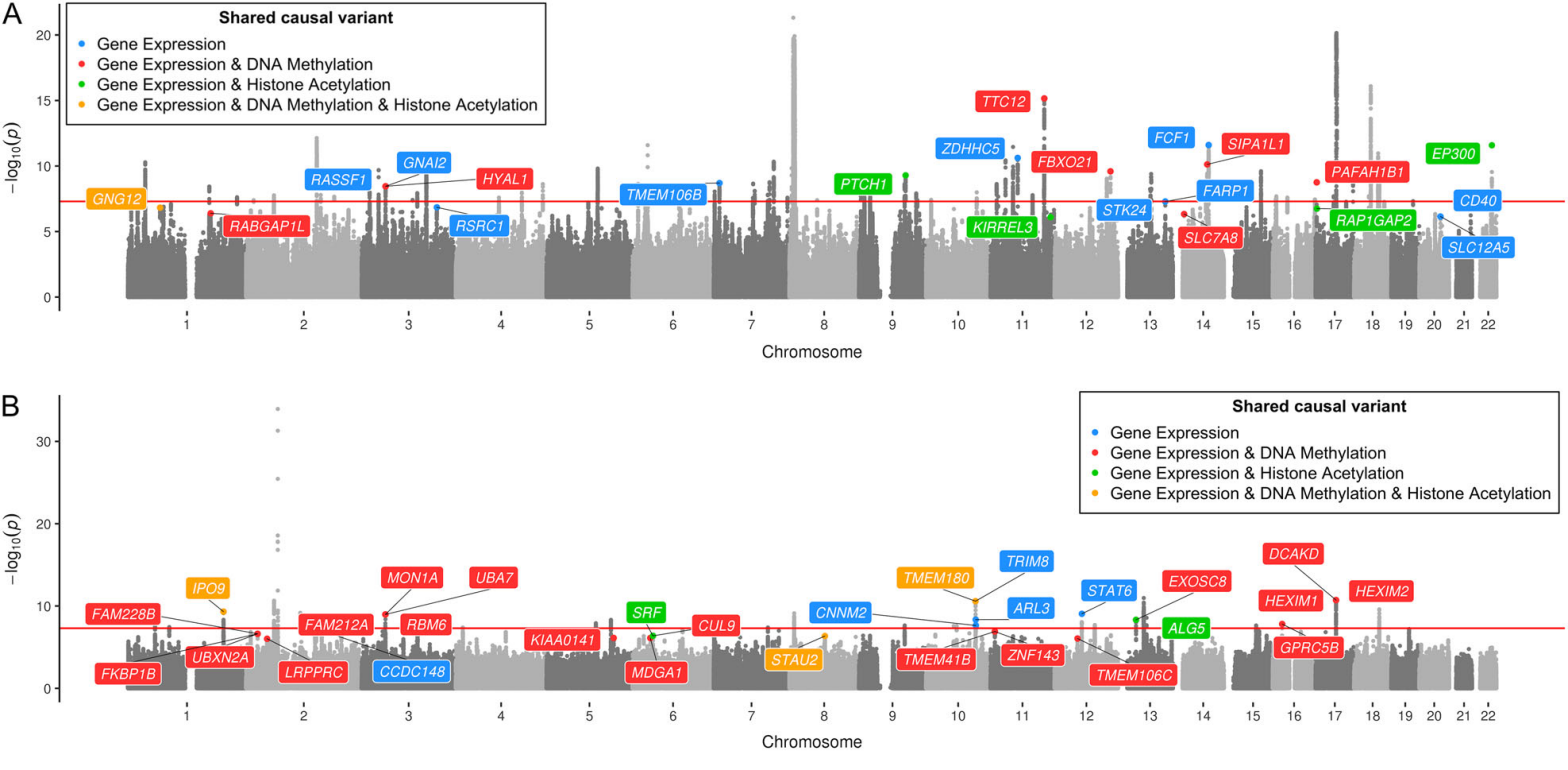
Manhattan plots highlighting loci with evidence of genetic colocalization. Manhattan plots for **a** neuroticism and **b** insomnia. Shared causal variants with traits are represented for the following scenarios: gene expression (blue), gene expression and DNA methylation (red), gene expression and histone acetylation (green) and gene expression, DNA methylation and histone acetylation (yellow). For example, in **a** *GNG12* is shown in yellow representing a case where there is a common causal variant (CCV) for neuroticism risk, expression of the gene *GNG12*, DNA methylation and histone acetylation at this locus. *PAFAH1B1* is shown in red, indicating that there is a CCV for neuroticism risk, expression of the *PAFAH1B1* gene and DNA methylation, but not for histone acetylation at this locus. The genome-wide significance threshold (*P* < 5 × 10^−08^) is illustrated in red

We applied the moloc method at loci with a trait-associated SNP (*P* < 5.0 × 10^−08^) using findings from five large-scale GWAS^16–19^ and molecular datasets (eQTL, mQTL and haQTL) derived from the brain tissue^14^. Across the five complex traits, we identified a total of 66 colocalization associations with GWAS loci and gene expression (Supplementary Tables 2–6). Of these, 40 provided evidence of colocalization with an epigenetic trait also. Altogether, four genetic loci colocalized with a complex trait and all three of the molecular phenotypes (gene expression, DNA methylation and histone acetylation). Figure 1 illustrates these associations for neuroticism and insomnia, whereas plots for the remaining traits can be located in Supplementary Figure 1.

We identified evidence of colocalization between complex and molecular traits at loci previously reported as well as novel findings. For example, we were able to replicate findings reporting that the expression of *KLC1* colocalizes with schizophrenia risk and DNA methylation^13^ (combined PPA = 97.9%). There were several other loci associated with schizophrenia that have been previously reported to colocalize with molecular traits (such as *CNNM2* and *PRMT7*^13^), as well as a several other genes where epigenetic mechanisms have not been previously detected to play a role in schizophrenia risk (such as *TSNARE1* and *ADOPT1*) (Supplementary Table 3).

**Fig. 2:**
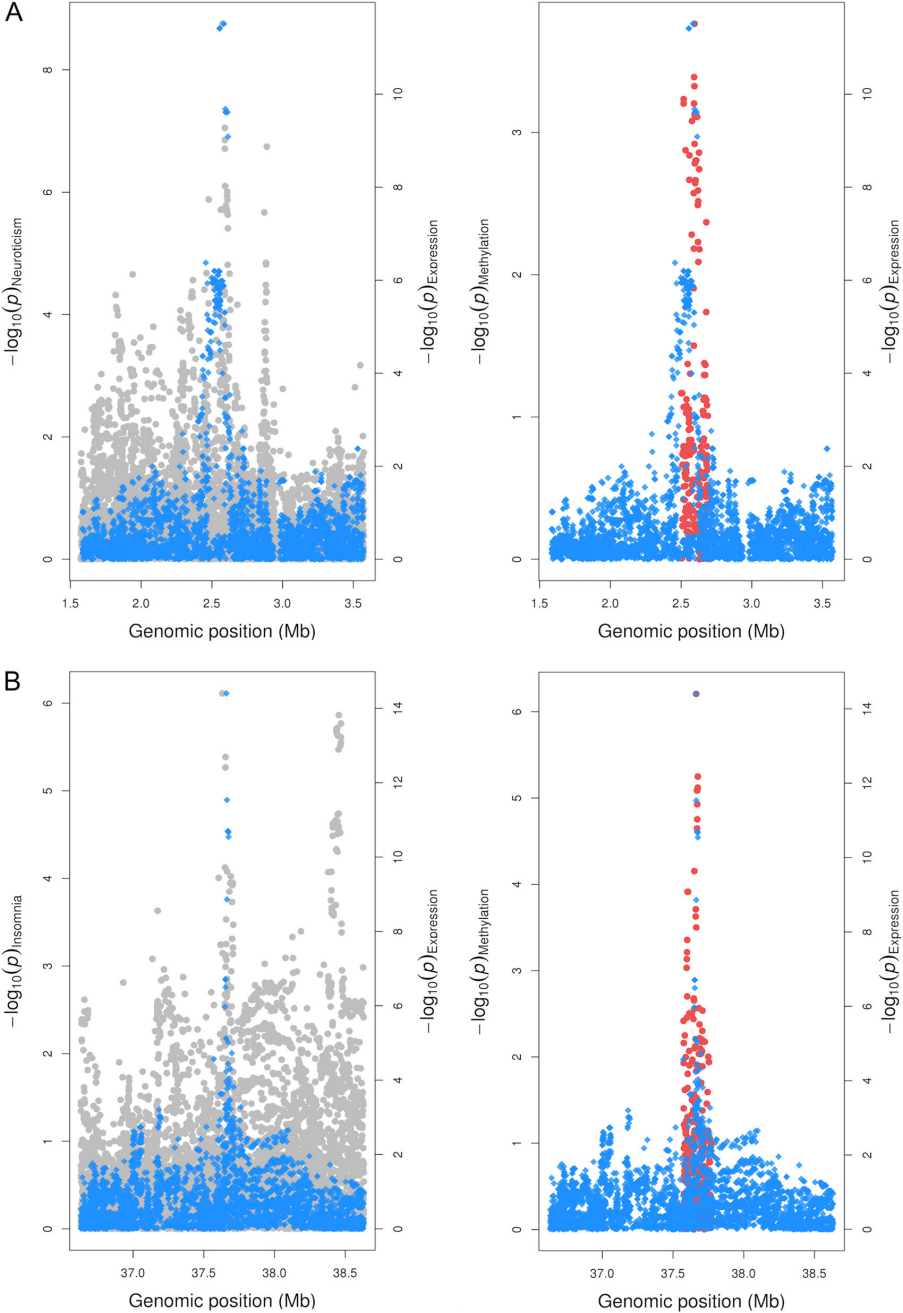
Regional association plots illustrating genetic colocalization at PAFAH1B1 and MDGA1. Regional association plots illustrating colocalizations for the *PAFAH1B1* gene (**a**) and *MDGA1* gene (**b**) with neuroticism and insomnia, respectively. Effects for genetic variants on complex traits (plotted in grey) and gene expression (blue) were available within a 1 Mb distance of the lead variant at each locus, whereas effects on DNA methylation levels (red) were confined to a 5kb distance

There were also novel associations with molecular phenotypes amongst the other complex traits. For instance, we uncovered evidence suggesting that neuroticism, gene expression and DNA methylation shared a CCV at the *PAFAH1B1* locus (combined PPA = 89.9%). Figure 2a illustrates the overlapping distributions of effects on each of these traits for variants at this region. We also observed evidence of colocalization for several genes at the *APOE* locus that were associated with Alzheimer’s disease. This included *TOMM40*, where results suggested that there was also evidence of colocalization with DNA methylation (combined PPA = 99.3%). However, given the extensive LD at this region, findings should be interpreted with caution^31^.

### Elucidating novel genes that may influence complex traits

We also applied our analytical pipeline to uncover potentially novel loci using a less stringent threshold (*P* ≤ 1.0 × 10^−06^). In this analysis, we identified 52 loci where complex traits and gene expression share a CCV, of which 33 provided evidence that these variants may also influence epigenetic traits (Fig. 1; Supplementary Figure 1). Our incentive for undertaking this analysis was that GWAS analyses may not identify evidence of association using observed effects on complex traits alone. However, by integrating evidence that SNPs at these loci also influence molecular traits derived from a relevant tissue type, we aimed to uncover novel loci in disease/trait variation. Table 1 provides an overview of the number of associations detected in our analysis.

**Table 1:** Number of associations with evidence of colocalization between complex and molecular traits

Complex trait	Gene expression	Gene expression and DNAm	Gene expression and histone acetylation	Gene expression and DNAm and histone acetylation
Loci meeting GWAS threshold				
Neuroticism	5	5	2	0
Schizophrenia	2	7	0	1
Education attainment	9	7	1	1
Alzheimer’s disease	6	3	1	0
Insomnia	4	9	1	2
Loci not meeting GWAS threshold				
Neuroticism	5	2	2	1
Schizophrenia	9	6	4	0
Education attainment	3	3	3	0
Alzheimer’s disease	1	0	0	0
Insomnia	1	10	1	1

The total number of associations detected in our analysis with evidence of colocalization as assessed by a posterior probability of association ≥80% using multiple-trait colocalization. Results are stratified based on the combination of molecular traits, which provided the strongest evidence that they share a causal variant with the associated complex trait

*GWAS* genome-wide association studies, *DNAm* DNA methylation

As an example of this, there was evidence that insomnia risk and molecular traits share a CCV at the *MDGA1* locus (combined PPA = 85.8%). However, the *P* value for the lead SNP at this region did not reach conventional GWAS thresholds (*P* = 7.7 × 10^−07^), suggesting that it would have been potentially overlooked based on GWAS evidence alone. As a validation of this finding, we found that a recent GWAS of insomnia with a larger sample size has found strong evidence of association at the *MDGA1* locus, which survives conventional corrections (*P* = 4.0 × 10^−12^)^32^. Figure 2b illustrates the overlapping distribution of effects for genetic variants at *MDGA1* on insomnia, gene expression and DNA methylation.

We identified several other instances from our analysis of loci with evidence of colocalization that have recently been detected by GWAS, suggesting that our analytical pipeline is valuable in terms of detecting novel findings. For example, we found that expression of the *CD40* and *SLC12A5* genes colocalize with risk of neuroticism. Both genes have subsequently been identified as associated with neuroticism at genome-wide significance in a GWAS meta-analysis^33^. Additionally, a recent large GWAS of educational attainment identified several genetic variants not previously found to reach genome-wide significance that we found to colocalize with molecular traits for the following genes: *DNAJB4*, *RERE*, *Corf73*, *DHX30*, *CD164* and *GLCC11*^34^.

### Pathway and tissue-specific enrichment analysis

Pathway analysis was conducted using ConsensusPathDB^24^ to investigate whether any sets of genes for each complex trait and disease reside along the same biological pathway (Supplementary Table 12). Amongst findings there was evidence that genes associated with neuroticism in our analysis (*SLC12A5*, *GNAI2* and *GNG12*) reside on the GABAergic synapse pathway (enrichment *P* = 2.34 × 10^−4^).

Our tissue-specific analysis indicated that various genes with evidence of colocalization are predominantly expressed in the brain. *SLC12A5*, *KLC1* and *KIRREL3* are expressed specifically in the cerebral cortex using data from the Human Protein Atlas^25^, whereas *MDGA1* was strongly expressed within the brain tissue using data from the GTEx^26^ project. *RAP1GAP2* was predominantly expressed within the cortex tissue using findings from the Mouse ENCODE project, amongst other loci are enriched in the brain tissue based on this dataset (Supplementary Table 13, Supplementary Figure 2).

We observed enrichment of an inverse relationship between DNA methylation and gene expression across loci, which provided evidence of colocalization for these molecular traits (*P* = 1.98 × 10^−03^), supporting previous evidence implicating DNA methylation as a transcriptional repressor^35^ (Supplementary Table 14). This effect appeared to be driven by CpG sites located near the transcription start site of genes, as 11 of the 13 sites located at these regions were inversely correlated with gene expression (84.6%). Performing the same analysis except with gene expression and histone acetylation suggested that there was weak evidence of enrichment for a directional relationship (*P* = 0.37). The regulatory annotations within brain tissue datasets for lead SNPs and CpG sites can be found in Supplementary Tables 15–17.

## Discussion

In this study, we have conducted an integrative analysis of GWAS and molecular QTL data to uncover mechanistic insight into the biological pathways underlying complex traits. We identified 118 colocalization associations between complex traits and gene expression, with 73 of these associations additionally colocalizing with proximal DNA methylation and/or histone acetylation in the brain tissue. Out of the 118 associations, 52 were potentially novel loci, which did not meet genome-wide significance corrections, but colocalized with molecular traits. Notably, several of these potentially novel loci have recently been validated by larger GWAS^32–34^, suggesting that other findings in our study are likely to be identified by GWAS as study sizes increase. Our findings should help future studies prioritize candidate genes and putative epigenetic mechanisms for functional follow-up analyses.

### Validation of previous findings

Applying our analysis pipeline to GWAS loci associated with complex traits and disease (i.e. *P* < 5 × 10^−08^) replicated previous findings reported by functional studies. For instance, our findings are consistent with an in-depth evaluation of the *KLC1* locus^3^. Variation at *KLC1* provided strong evidence of colocalization in our study (combined PPA = 97.9%), where the highest individual posterior probability suggested that both gene expression and DNA methylation may be involved along the causal pathway to schizophrenia risk. This result also supports findings from an epigenome-wide association study implicating DNA methylation as potentially playing a role in schizophrenia risk at this locus^36^. Furthermore, Hi-C interactions have been identified at the promoter region of *KLC1* within the brain tissue, which further helps validate the putative regulatory mechanism implicated by our results^37^.

Amongst other established GWAS loci, there was evidence suggesting that expression of the *TOMM40* gene and DNA methylation may play a role in Alzheimer’s disease. An exploratory analysis has found that regulatory element methylation levels in the *TOMM40-APOE-APOC2* gene region correlate with Alzheimer’s disease^38^. However, there is also evidence that, although SNPs at this region are known to influence Alzheimer’s disease, gene expression, and DNA methylation, they may be attributed to different causal variants^39^. Moreover, there is a complex LD structure at this region^31^, suggesting that further analysis is required to fully understand the mechanisms underlying this association.

In cases where gene expression was found to colocalize with DNA methylation, we observed evidence of enrichment for an inverse relationship between these molecular phenotypes. Such inverse correlations support established biology that DNA methylation plays a role in silencing gene transcription^40^. However, recently there has been conflicting reports concerning whether DNA methylation on its own is sufficient to lead to transcriptional repression of promoters^35,41^. Further analysis investigating the epigenetic mechanisms identified by our study should prove valuable in fully understanding the role of DNA methylation in gene regulation.

### Novel leads

We were also able to identify evidence of colocalization at GWAS loci that have not been linked previously by functional analyses or integrative studies harnessing molecular traits. For instance, the underlying biology explaining a GWAS association with neuroticism on chromosome 17 (lead SNP = rs12938775) has yet to be thoroughly evaluated. Our findings suggest that *PAFAH1B1* may be the likely causal gene at this locus, as well as implicating the involvement of DNA methylation along the causal pathway to neuroticism susceptibility as well (combined PPA = 90.0%). *PAFAH1B1* (also known as *LIS1*) is involved in neuronal migration, the process by which different classes of neurons are brought together so that they can interact appropriately^42^. Functional evaluations of how changes in DNA methylation may influence neurological function at loci such as this may prove valuable in understanding epigenetic contributions to disease susceptibility. Moreover, doing so will help improve the accuracy of early disease prognosis.

As well as helping characterize associations detected by GWAS studies, we have also uncovered evidence for many novel genes, which may influence complex trait variation and therefore represent promising candidates for future endeavours. The association with insomnia risk at the *MDGA1* locus is an example of this, particularly given that it has recently been validated by a large-scale GWAS^32^. Furthermore, our results may provide functional insight into this association, by suggesting that *MDGA1* may be the responsible causal gene and that DNA methylation may also play a role in disease risk at this locus (combined PPA = 85.8%). Similar to *PAFAH1B1*, *MDGA1* has also been report to play a role in neuronal migration^43^ and based on our tissue-specific analysis is predominantly expressed in the brain tissue.

*SLC12A5*, associated with neuroticism in our analysis (combined PPA = 99.0%), was amongst other promising candidates which has yet to be discovered by GWAS. This gene encodes the neuronal KCC2 channel, which plays a crucial role in fast synaptic inhibition^44^. *SLC12A5* was also amongst the genes associated with neuroticism in our analysis that resides along the GABAergic synapse pathway (along with *GNAI2* and *GNG12*). A recent study has suggested that GABAegic neurons are causally associated with risk of bipolar disorder^45^, a condition previously linked with higher global measures of neuroticism^46^. The association between *KIRREL3* and neuroticism (combined PPA = 92.2%) is another finding that has yet to be identified by GWAS, which warrants in-depth functional evaluation. *KIRREL3* regulates target-specific synapse formation and has been previously linked with neurodevelopmental disorders^47^. Our tissue-specific analysis suggests that both *SLC12A5* and *KIRREL3* are predominantly expressed in the brain tissue.

### Limitations of this study

In terms of limitations of this study, we recognize that integration of GWAS results with QTL data is limited by the sample sizes used to derive summary statistics, which is particularly noteworthy for QTL data available in the brain. It may be the case that replication in blood can provide greater power due to the larger sample sizes available. It has been shown that top *cis*-eQTL and mQTL are highly correlated between blood and brain tissues^48^. Future work could take advantage of this correlation and the higher power in these blood datasets.

There is also evidence that the expression of certain genes is both highly tissue and disease specific^45^. Recently, it has been shown that both tissue-specific and tissue-shared eQTL provide a substantial polygenic contribution to various complex traits^27^. Further investigation into the tissue specificity of our results could be interesting since the ROSMAP/Brain xQTL^14^ dataset comes specifically from the dorsolateral prefrontal cortex region of the brain. Analysis of effects in other regions of the brain may be interesting to potentially identify disease-relevant regions. We were also limited as the mQTL data was confined to 5 kb windows affecting the coverage we could get within a genomic region. Whilst the nature of this mQTL dataset means we may have missed some true effects, it also means we are unlikely to have identified false positives. It is also worth noting that as the number of molecular studies increases, so too does the likelihood of detecting incidental QTL-GWAS overlaps^3^. Hence, developments concerning robust methods in colocalization should prove to be extremely valuable and important for future research.

## Conclusions

By integrating GWAS findings with data concerning brain cortex-derived molecular phenotypes, we have helped uncover putative epigenetic and transcriptomic drivers of complex traits and disease. The polygenic nature of many psychiatric disorders has led to many challenges in terms of developing novel therapeutics^49^. Our work has focused on the prioritization of GWAS hits to uncover loci, which may be potential targets for therapeutic intervention. Genetically informed targets have been shown to have a higher success rate in clinical development, with such targets being more likely to progress to phase III trials^15^.

Animal studies are one possible approach to validate and help translate the findings of our study. Knockout studies, for example, could be used to investigate the impact of whole gene loss-of-function on a particular phenotype^50^. Furthermore, the loci we have uncovered associated with changes in DNA methylation could be validated by studies interested in using this epigenetic marker to help predict later life disease events. Such endeavours may help elucidate CpG sites that may be valuable for disease prediction and patient stratification.

## Supplementary information

Supplementary Figures.

Supplementary Tables.
