# Supplementary material for: Leveraging brain cortex-derived molecular data to elucidate epigenetic and transcriptomic drivers of complex traits and disease
Source: Transl Psychiatry. 2019 Feb 28;9:105. doi: 10.1038/s41398-019-0437-2 (PMC6395652; doi:10.1038/s41398-019-0437-2)

B

A

C


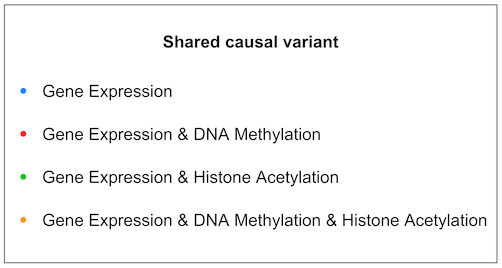

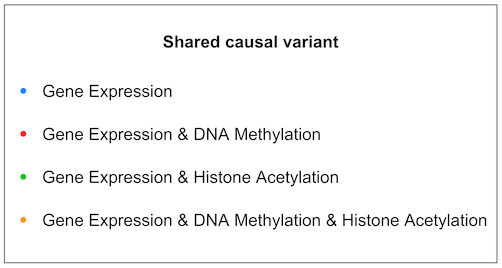

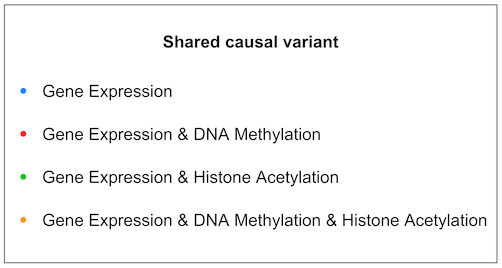


**Supplementary Figure 1 |** Manhattan plots for (A) Schizophrenia, (B) Educational Attainment and (C) Alzheimer’s Disease. Shared causal variants with traits are represented for the following scenarios; Gene expression (blue), Gene expression & DNA methylation (red), Gene expression & histone acetylation (green) and Gene expression & DNA methylation & histone acetylation (yellow). The genome-wide significance threshold (P < 5 x 10^-08^) is shown in red.

**Supplementary Figures 2:** Heatmaps illustrating tissue-specific expression of genes which are predominantly expressed in brain tissue:


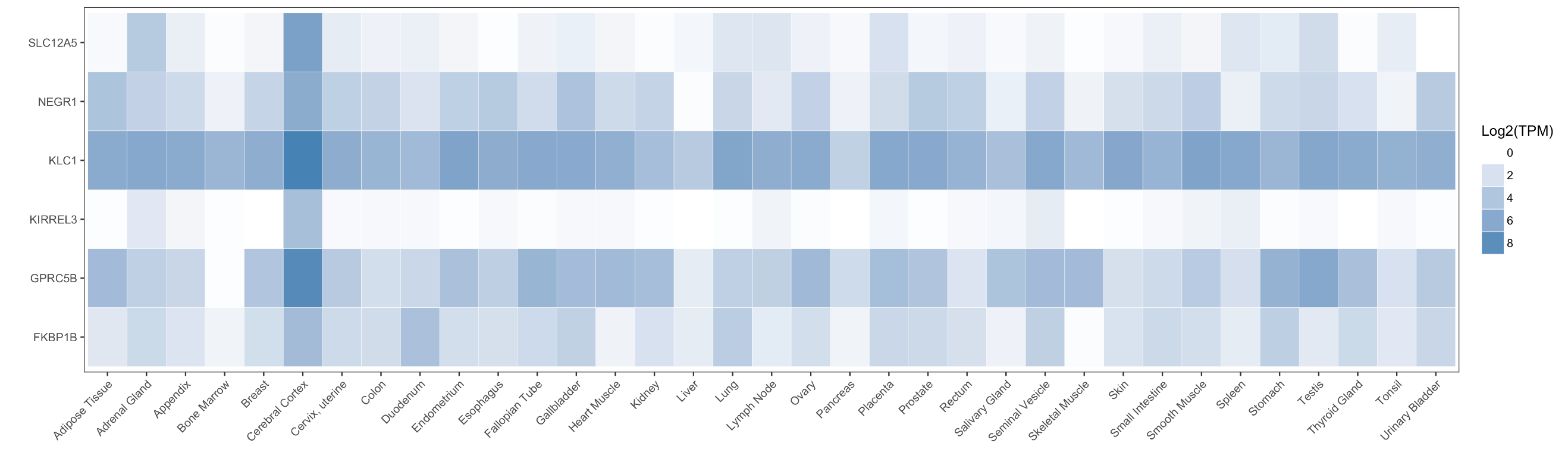
Human Protein Atlas:


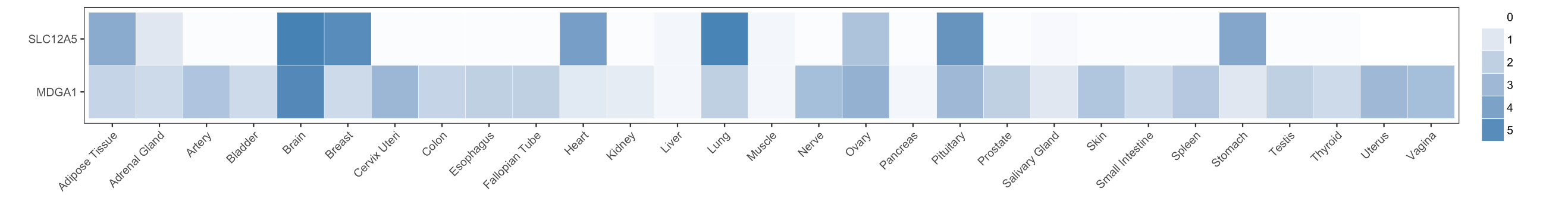
GTEx:

Mouse ENCODE project (Cerebellum):


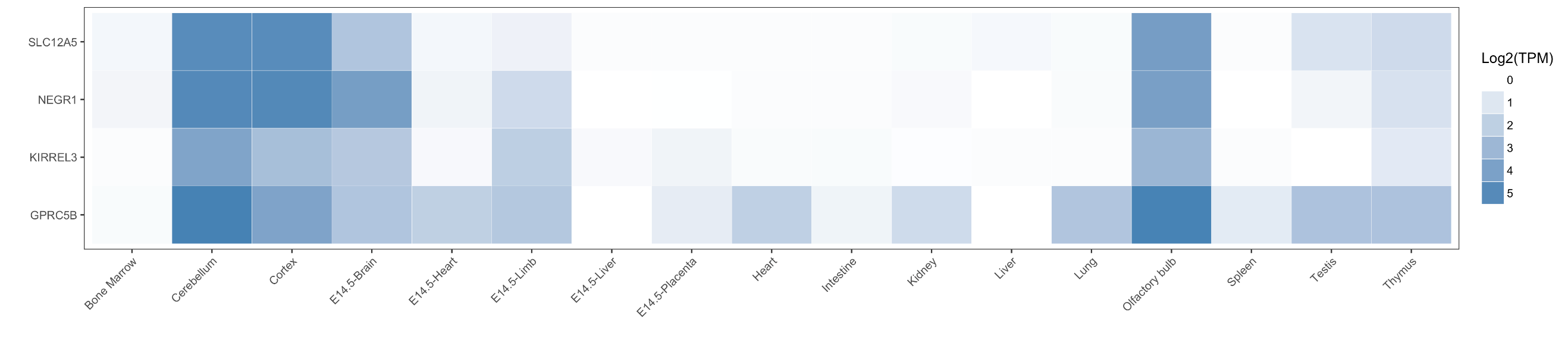


Mouse ENCODE project (Cortex):


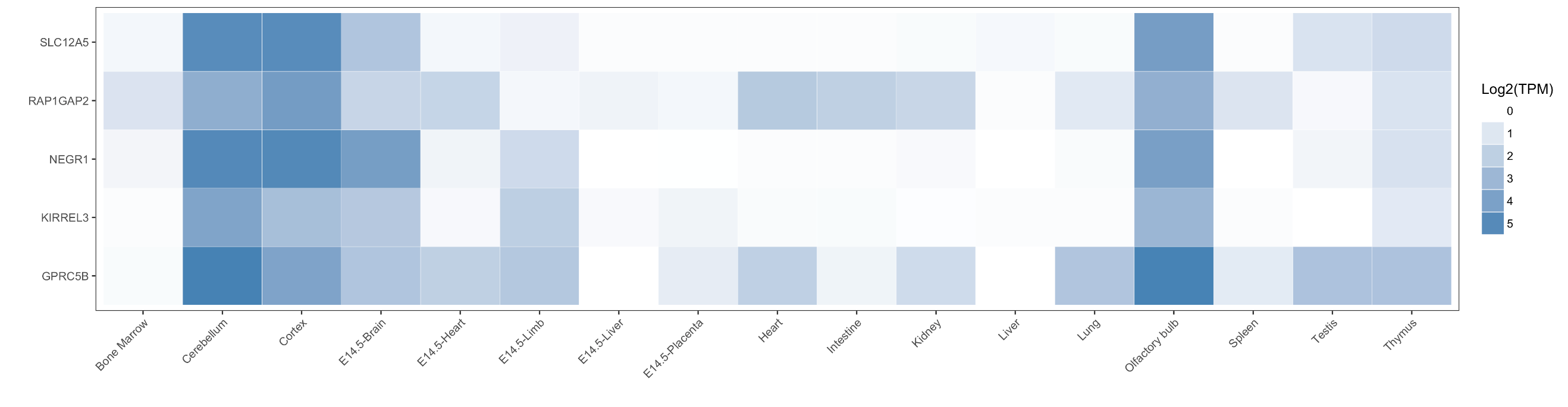


Mouse ENCODE project (E14.5 Brain):


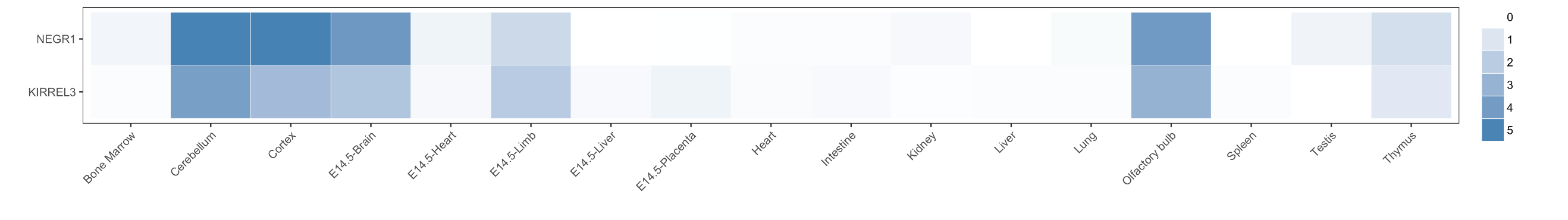

Supplement: Supplementary file 1 — Supplementary Figures. [file 41398_2019_437_MOESM1_ESM.docx]
